# Cobalt(II) and Cadmium(II) Metal–Organic Framework with Benzene-1,3,5-tricarboxylate and Viologen Guest: Stimuli-Responsive Photochromism, Volatile Amine Detection, and Hydrogen Generation

**DOI:** 10.1021/acsomega.5c01484

**Published:** 2025-04-17

**Authors:** Ferihan Tataş Coşkun, Kutalmış Gökkuş, Okan Zafer Yeşilel

**Affiliations:** † Department of Chemistry, Faculty of Science, Eskişehir Osmangazi University, Eskişehir 26480, Türkiye; ‡ Department of Environmental Engineering Faculty of Engineering and Architecture, Kastamonu University, Kastamonu 37500, Türkiye

## Abstract

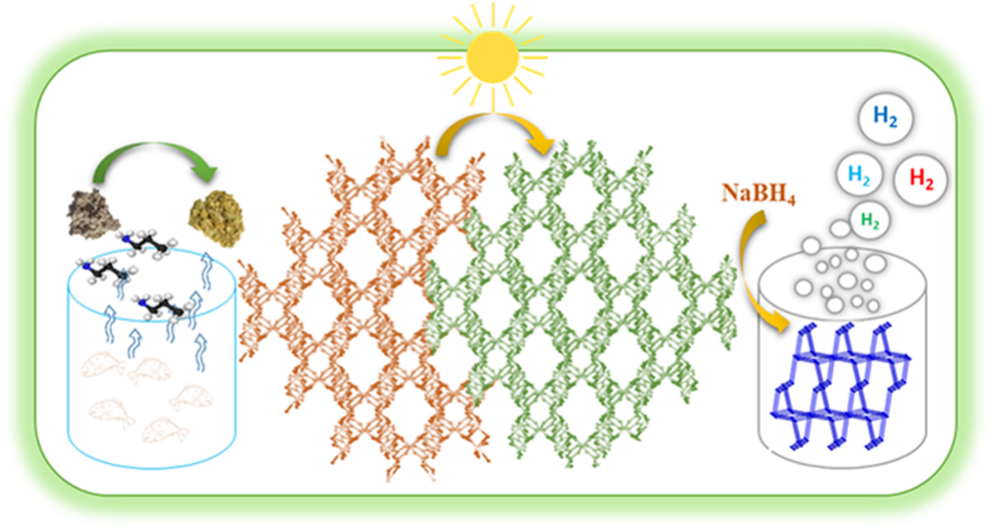

Two Co(II) and Cd(II) coordination polymers, {(Me_2_bipy)[Co_5_(btc)_3_(Hbtc)(OH)]·2DMF·9H_2_O}*_n_***(CoMOF)** and {(Me_2_bipy)[Cd_2_(μ_4_-btc)_2_]}*_n_* (**OGU-2**), were synthesized and characterized using IR spectroscopy, elemental analysis, and powder and single-crystal X-ray diffraction techniques. The Cd1 ion is seven-coordinated, adopting a distorted monocapped trigonal prismatic geometry (CdO_7_). **OGU-2** exhibits a 3D porous framework with a rare flu-3,6-*C*2/*c* topology. The framework contains one-dimensional channels along the *c* axis with dimensions of approximately 10 × 10 Å^2^. The photochromic behavior of **OGU-2** was investigated, revealing excellent chromic properties in response to multiple external stimuli, including UV light, temperature, and organic amines. **CoMOF**, a coordination polymer reported previously, along with **OGU-2**, was evaluated as a catalyst for hydrogen gas production via the methanolysis reaction of NaBH_4_. The results demonstrated that these coordination polymers exhibited high catalytic activity for hydrogen production, comparable to the high values reported in the literature.

## Introduction

Materials that respond to external stimuli by changing color offer significant opportunities for applications in various innovative fields.^1^ Among these materials, viologens stand out as a remarkable group.^1−4^ Viologen derivatives (1,1′-disubstituted-4,4′-bipyridinium) can generate radicals through electron transfer (ET) in response to external stimuli such as pH, heat, light, electricity, solvents, and organic amines.^2,5−7^ This process enables visible and reversible color changes. Due to these properties, viologens are used in numerous high-tech applications, including electrochromic devices, photoresponsive displays, sensors, optical switches, smart windows, and ink-free erasable printing.^6,8−10^

Despite their potential, current viologen-based materials typically respond to only a single stimulus, limiting their application scope.^11^ Moreover, the slow response speed and lack of stability under external stimuli represent significant drawbacks. Developing fast, stable, and multi-stimuli-responsive viologen-based materials is, therefore, crucial for practical applications. A promising strategy to achieve this involves incorporating viologen derivatives into the porous structures of metal–organic frameworks (MOFs), which are highly stable and feature high surface areas and electron-donating groups.^7,9,12,13^ Specifically, uncoordinated carboxylate oxygens within MOF structures can interact with viologen groups, facilitating electron transfer and enhancing chromic properties.^1,7,13^

Although amines are widely used in the polymer, paint, and pharmaceutical industries, their toxic and corrosive properties can cause significant harm to the skin, eyes, and respiratory system.^14^ The detection of volatile amines is of great importance, as it plays a vital role in ensuring industrial safety and protecting environmental health by identifying potentially harmful substances that could pose risks to human health and the ecosystem.^14,15^ The fact that volatile amines are typically colorless complicates their visual detection. Conventional detection methods, such as high-performance liquid chromatography (HPLC), fluorometric analysis, gas chromatography–mass spectrometry (GC-MS), and cyclic voltammetry, are often expensive and unsuitable for on-site use. Consequently, chromic materials that undergo color changes upon contact with amines offer a better alternative for the rapid, economical, and visual detection of these compounds.

Global warming has been one of the most important threats to the future of the world for many years. The most important cause of global warming is the burning of fossil fuels. Although various strategies have been developed to reduce the impact of fossil fuels, the best approach among them is the use of alternative energy sources. In this respect, hydrogen energy is one of the most important alternatives for the future due to its complete environmental friendliness and high energy density (142 kJ mol^–1^).^16,17^ However, because hydrogen energy is quite new, especially compared to wind and solar energy, there are still serious difficulties in terms of the production, storage, and transportation of hydrogen.^18^

One of the biggest challenges for hydrogen energy is the production of hydrogen gas with high purity and efficiency. Metal hydrides, in particular, stand out in hydrogen production due to their potential use in fuel cells. For example, NaBH_4_ has advantages such as high chemical stability, nontoxicity, environmentally friendly structure, nonvolatile nature, frequent preference as a hydrogen carrier, and the ability to recycle the products formed as a result of the reaction.^19,20^ H_2_ gas is easily produced from NaBH_4_ by hydrolysis or alcoholysis. However, since both processes are quite slow, catalysts are needed to accelerate the process. Therefore, there are many examples in the literature where different catalysts are produced.^17,21−23^ Despite these efforts, the need for the development of new catalysts continues.

In this study, viologen-based 3D MOFs, {(Me_2_bipy)[Co_5_(btc)_3_(Hbtc)(OH)]·2DMF·9H_2_O}*_n_* (**CoMOF**)^10^ and {(Mebipy)[Cd_2_(μ_4_-btc)_2_]·8H_2_O}*_n_* (**OGU-2**), were successfully synthesized using 1,1′-dimethyl-4,4′-bipyridinium bromide and benzene-1,3,5-tricarboxylic acid (BTC). The structure of **OGU-2** was characterized using FT-IR, elemental analysis, and single-crystal X-ray diffraction (SC-XRD) techniques. Additionally, its photochromic and volatile amine sensing properties were investigated. The phase purity and thermal stability of **OGU-2** were determined using thermal analysis techniques (TG/DTA) and powder X-ray diffraction (PXRD), respectively. The photochromic and vapochromic properties of **OGU-2** were investigated. The mechanism underlying its color change was further examined through electron spin resonance (ESR) analysis.

## 2 Material and Methods

### 2.1 Materials and Measurements

All chemicals used were analytical reagents and purchased commercially. The IR spectra were recorded with a Bruker Tensor 27 FT-IR spectrometer using KBr pellets in the range 400–4000 cm^–1^. Elemental analyses (C, H, and N) were performed with a PerkinElmer 2400C Elemental Analyzer. Thermal analyses (TG/DTA) were performed with a PerkinElmer Diamond TG/DTA Thermal Analyzer at a heating rate of 10 °C min^–1^ in a static atmosphere in the temperature range of 30–1000 °C. A Panalytical Empyrean X-ray diffractometer with Cu Kα radiation was used to record powder X-ray diffraction patterns. Electron spin resonance (ESR) spectrum was recorded on a JEOL/JESFA-300 spectrometer at room temperature. A photochromic test was performed using a 300 W Hg lamp. The diffuse reflectance spectra were carried out using a Shimadzu UV-2600 spectrophotometer and BaSO_4_ as a 100% reflectance standard in the wavelength range 200–800 nm. The ^1^H NMR spectrum was taken on a JEOL ECZ 500R spectrometer at room temperature.

A Bruker Smart Apex II CCD diffractometer outfitted with a Mo Kα radiation (λ = 0.71073 Å) was used for single-crystal X-ray diffraction data collection. SHELXT-2015 program in conjunction with the OLEX2 was used to solve the structures with direct methods and the structure refinements were carried out by full-matrix least-squares on all *F*^2^ data using SHELXL-2015.^24^ In the compounds, all hydrogen atoms were calculated and refined using riding and free modes. The crystal structures were drawn using the Mercury program.^25^ ToposPro software was used for topological analysis of the compounds.^26^ Details of crystal data, data collection, structure solution, and refinement are given in Table S1.

### 2.2 Synthesis of the Compounds

1,1′-Bis(carboxymethyl)-4,4′-bipyridinium bromide was synthesized according to the literature.^27^ Me_2_bipy was synthesized *in situ* via the decarboxylation of 1,1′-bis(carboxymethyl)-4,4′-bipyridinium.^4^

#### {(Me_2_bipy)[Cd_2_(μ_4_-btc)_2_]}_*n*_ (**OGU-2**)

A mixture of Cd(NO_3_)_2_·4H_2_O (0.1 mmol, 30.8 mg), H_3_btc (0.1 mmol, 21 mg), and 1,1′-bis(carboxymethyl)-4,4′-bipyridinium bromide (0.1 mmol, 43.4 mg) was dissolved in a mixture of 9 mL of DMF and 3 mL of water. The solution was sonicated for 30 min, then the clear solution was transferred to a glass vial (25 mL), and the vial was kept at 80 °C for 72 h. After cooling to room temperature, orange crystals were obtained, filtered, washed with DMF/water mixture, and dried in air. Yield: 18.22 mg (75.2%). %. FT-IR (ν, cm^–1^): 3440, 3087, 2918, 1641, 1612, 1550, 1429, 1363 791, 709, 421 (Figure S1).

#### {(Me_2_bipy)[Co_5_(btc)_3_(Hbtc)(OH)]·2DMF·9H_2_O}*_n_* (**Co-MOF**)

**CoMOF** was synthesized according to the literature.^10^ It has been reported that the **CoMOF** was synthesized using a solvent mixture of DMF, water, and methanol at 85 °C. In this study, **CoMOF** was also synthesized using a DMF/water mixture. Despite slight differences in the crystal data, both methods yield the same material with minimal structural variations. FT-IR (ν, cm^–1^): 3431, 2909, 1624, 1435, 1381, 899, 752 (Figure S1). Powder X-ray diffraction (PXRD) analysis was performed to evaluate the phase purity of **Co-MOF**, and it was determined that the theoretical and experimental results are in agreement (Figure S2).

### 2.3 Hydrogen Production Experiments

The primary objective of the experiments was to evaluate the catalytic effectiveness of **OGU-2** and **Co-MOF** in hydrogen production via NaBH_4_ methanolysis. The water displacement method was utilized to measure hydrogen evolution. The reactions were conducted under controlled conditions at 30 °C with a stirring rate of 750 rpm, employing 0.125 g of NaBH_4_ as the hydrogen source, 0.01 g of catalyst (either **OGU-2** or **CoMOF**) to accelerate the reaction, and 10 mL of methanol as the reaction medium. The hydrogen generation rate (HGR) values were systematically analyzed by varying key parameters, including NaBH_4_ concentration, catalyst amounts, and reaction temperature, to determine the optimal conditions for each catalyst. Additionally, the activation energy for the methanolysis reaction was calculated by performing the experiments at different temperatures.

## 3 Results and Discussion

### 3.1 Crystal Structure

#### {(Me_2_bipy)[Cd_2_(μ_4_-btc)_2_]}*_n_*

**Figure 1:**
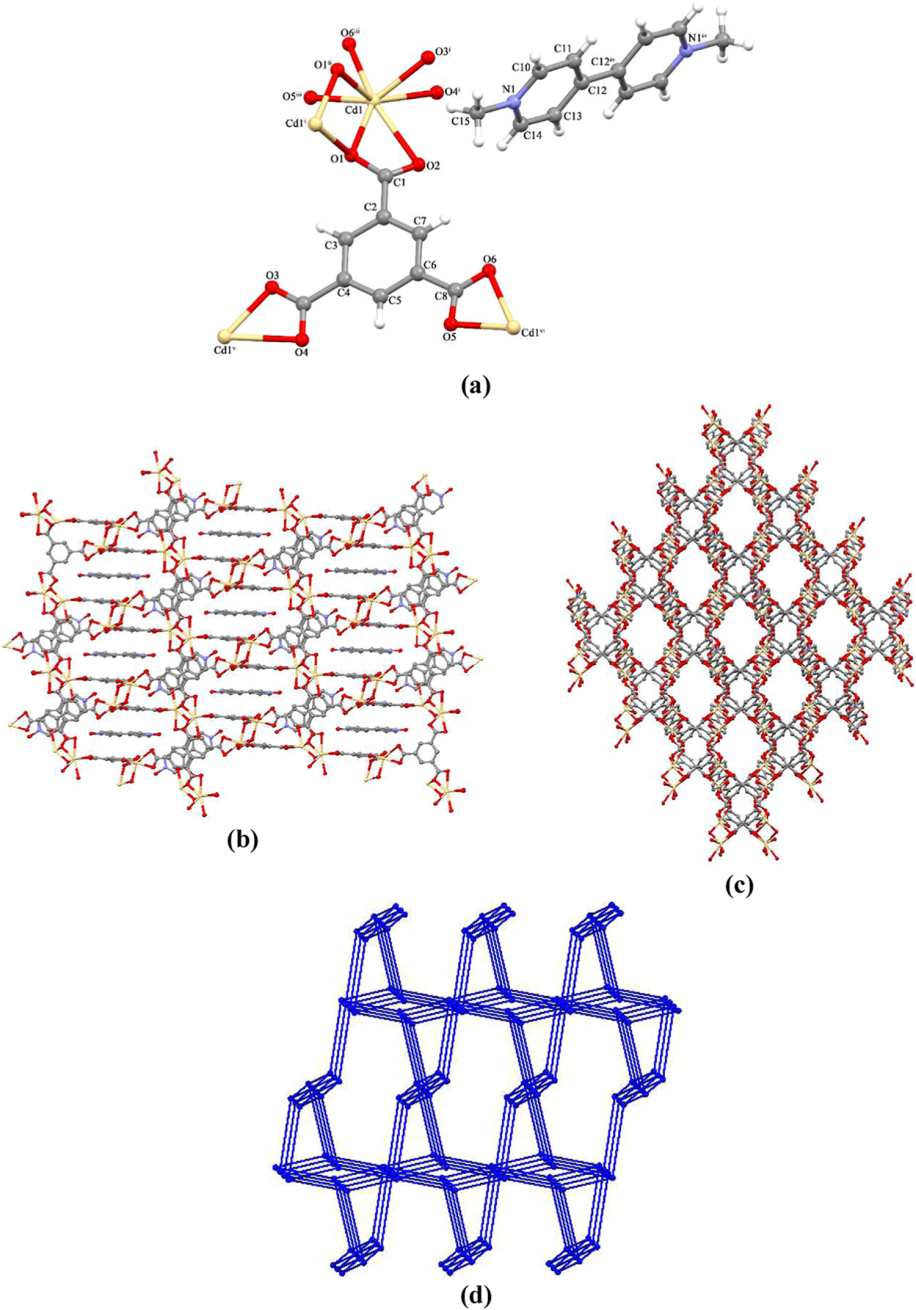
(a) Molecular structure of the **OGU-2**. (b) 3D framework of **OGU-2**. (c) 3D framework contains 1D channels. (d) View of flu-3,6-*C*2/*c* topology symmetry codes: (*x* + 1/2, −*y* + 3/2, *z* + 1/2; (ii) −*x* + 3/2, −*y* + 3/2, −*z* + 1; (iii) *x* + 1/2, *y* – 1/2, *z*; (iv) −*x* + 3/2, −*y* + 3/2, −*z* + 2; (v) *x* – 1/2, −*y* + 3/2, *z* – 1/2; (vi) *x* – 1/2, *y* + 1/2, *z*).

Single-crystal X-ray diffraction analysis reveals that **OGU-2** crystallizes in the monoclinic crystal system of the *C*2/*c* space group. The asymmetric unit of **OGU-2** consists of one Cd(II) ion, one btc ligand, and half of me_2_bipy ion. As shown in Figure 1a, Cd(II) ions adopt a distorted pentagonal bipyrimidal geometry containing seven oxygen atoms from four different btc ligands. The lengths of the Cd–O bonds fall in the range of 2.247(5)–2.611(6) Å (Table S2). Each btc ligand coordinates to four Cd(II) ions through three different carboxylate groups adopting heptadentate coordination modes. The binuclear clusters were connected by btc ligands into a 3D porous framework with 1D rhombus channels and double walls. (Figure 1c.). Moreover, π···π stacking interactions between the phenyl ring of btc (Cg1 = C2–C3–C4–C5–C6–C7) and the pyridine ring of the Me_2_bipy cation (Cg2 = N1–C10–C11–C12–C13–C14) are found in **OGU-**2, and the corresponding centroid distance (Cg1···Cg2) is 3.616(4) Å. This interaction further stabilizes the host–guest system. In addition, a PLATON program analysis shows that there is about 18.90% (616.6 Å^3^) of the total crystal volume accessible to solvents. Topologically, **OGU-2** has 3,6-connected two nodal nets with flu-3,6-*C*2/*c* topology (the point symbol of {4^2^.6}^2^{4^4^.6^2^.8^7^.10^2^}) (Figure 1d).^28^

### 3.2 Photochromism

MOFs containing viologen groups exhibit photochromic behavior. The nitrogen atoms in viologen groups are positively charged, leading to an electron deficiency in viologen compounds. Electron-rich atoms located within a close distance (less than 4 Å) to the positively charged nitrogen atoms in viologen groups transfer their electrons to the π* orbitals of viologens under UV light. This process results in the formation of viologen radicals. The radicals, stabilized by the aromatic rings in the bipyridine group, can absorb light at different wavelengths, leading to a color change in the MOF. In this study, we report the properties of an **OGU-2** with an anionic framework hosting methyl-viologen cations in its pores (Figure 1).

**Figure 2:**
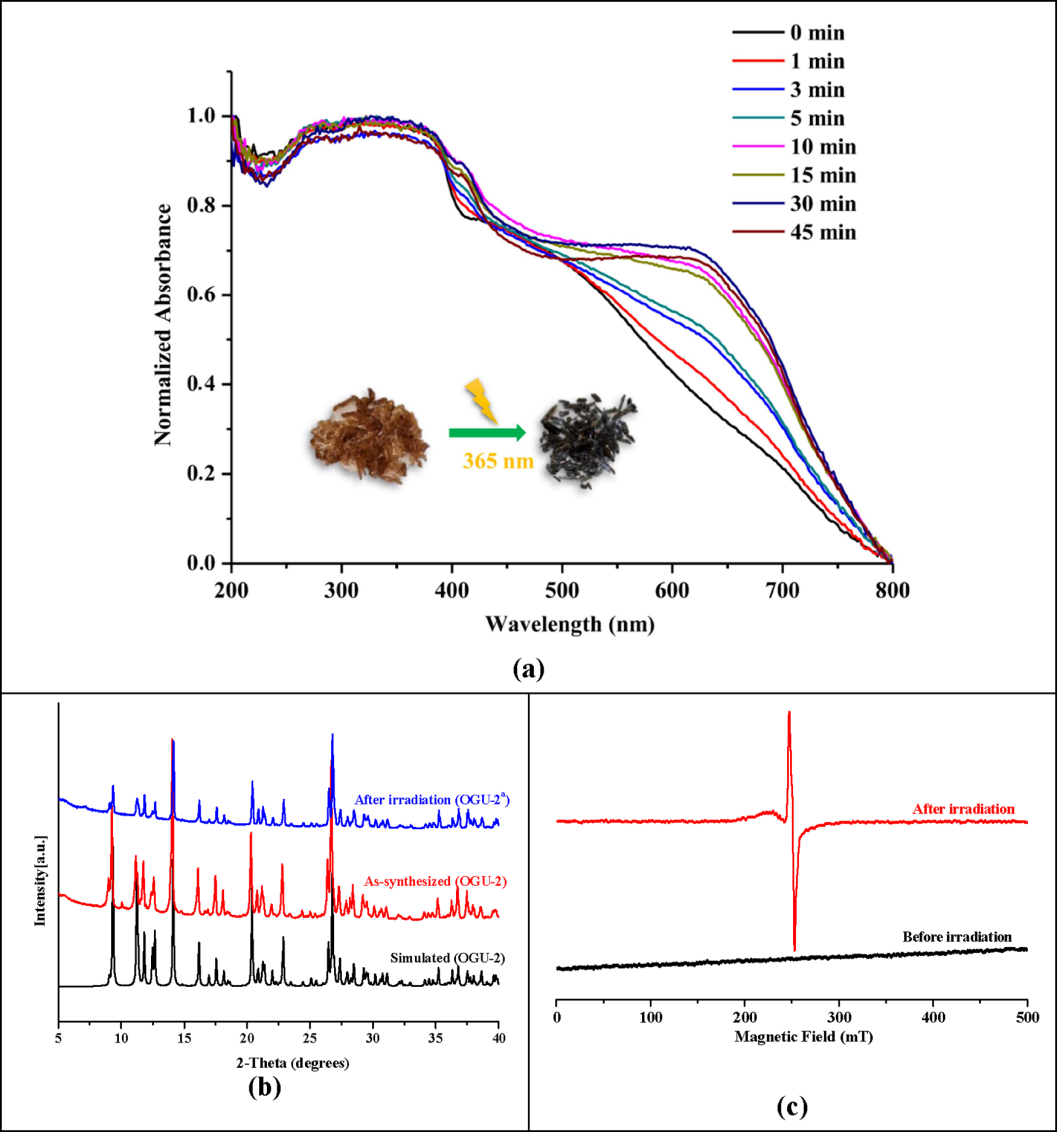
(a) Diffuse reflectance spectra of **OGU-2** and **OGU-2**^**a**^. (b) PXRD spectra of **OGU-2** and **OGU-2**^**a**^. (c) ESR spectra of **OGU-2** (black) and **OGU-2**^**a**^ (red).

**OGU-2** exhibits exceptionally fast photochromism. When exposed to a 365 nm xenon UV lamp, the crystals change color from orange to green, a transformation visible to the naked eye (Figure 2). Similarly, under sunlight, the MOF undergoes a comparable color change within approximately 10 s (Figure S5). This rate of photochromic response is significantly faster than other MOFs containing viologen units. As shown in Table S3, **OGU-2** undergoes a rapid color change from orange to green within approximately 10 s under sunlight. This response time is slightly slower than [Cd_2_(μ_4_-L)Cl_3_]*_n_*, which exhibits a similar transformation in just 3 s. The rapidity is attributed to the close proximity of the Me_2_bipy unit’s positively charged nitrogen atoms to the donor carboxyl oxygen atoms. Furthermore, the strong electron deficiency of the positively charged viologen groups within the pores enhances their interaction with the anionic MOF framework, facilitating the transfer of electrons to the viologens more efficiently.

Electron transfer is likely to occur from the oxygen atoms closest to the Me_2_bipy guest molecules (with a distance of 3.478 Å between them). When the green-colored **OGU-2**^**a**^ is kept in the dark for 1 day, it reverts to its original orange color. PXRD spectrum analysis revealed no structural changes compared to the original sample, confirming that the MOF exhibits rapid and reversible photochromism (Figure 2b). Electron spin resonance (ESR) and solid-state UV–vis spectroscopy measurements were performed to confirm the role of radicals in this photochromic process (Figure 2c). The dark green **OGU-2**^**a**^ obtained after irradiation exhibits a strong absorption band with a maximum at 644 nm in the visible region (Figure 2a). As shown in Figure 2c, the ESR spectrum presents a strong ESR signal with a g value of 2.0027, confirming the presence of photoinduced viologen radicals.

**Figure 3:**
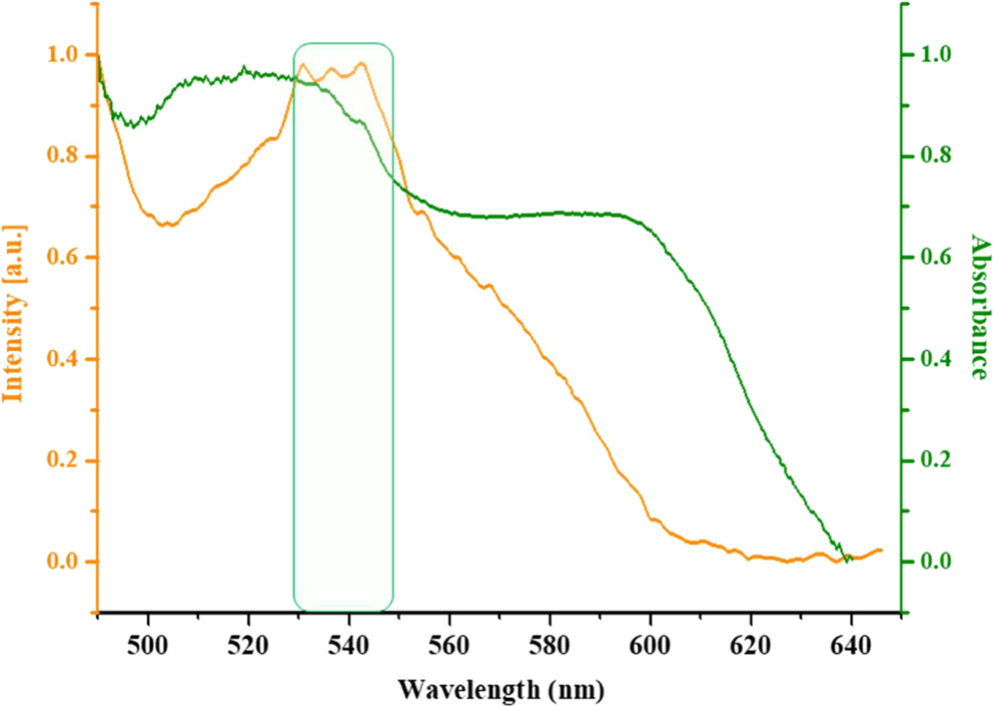
Energy transfer process of **OGU-2**. The orange line represents the emission spectrum, and the green line represents the UV–vis absorption spectrum of **OGU-2**.

The energy transfer process resulting from the interaction between OGU-2 and light was examined through spectroscopic analyses. The emission spectrum of OGU-2 prior to discoloration is depicted by a yellow line, whereas the UV–Vis absorption spectrum recorded after irradiation is represented by a green line (Figure 3).

The solid-state luminescence property of **OGU-2** was investigated at room temperature. Upon excitation at 460 nm, **OGU-2**^**a**^ exhibited emission at 540 nm (Figure 3). This emission peak partially overlapped with the absorption peak at 520 nm in the UV–vis spectrum of **OGU-2**^**a**^ after 45 min of light exposure, indicating an energy transfer process. These results are consistent with the literature.^7,29^

### 3.3 Vapochromism

**Figure 4:**
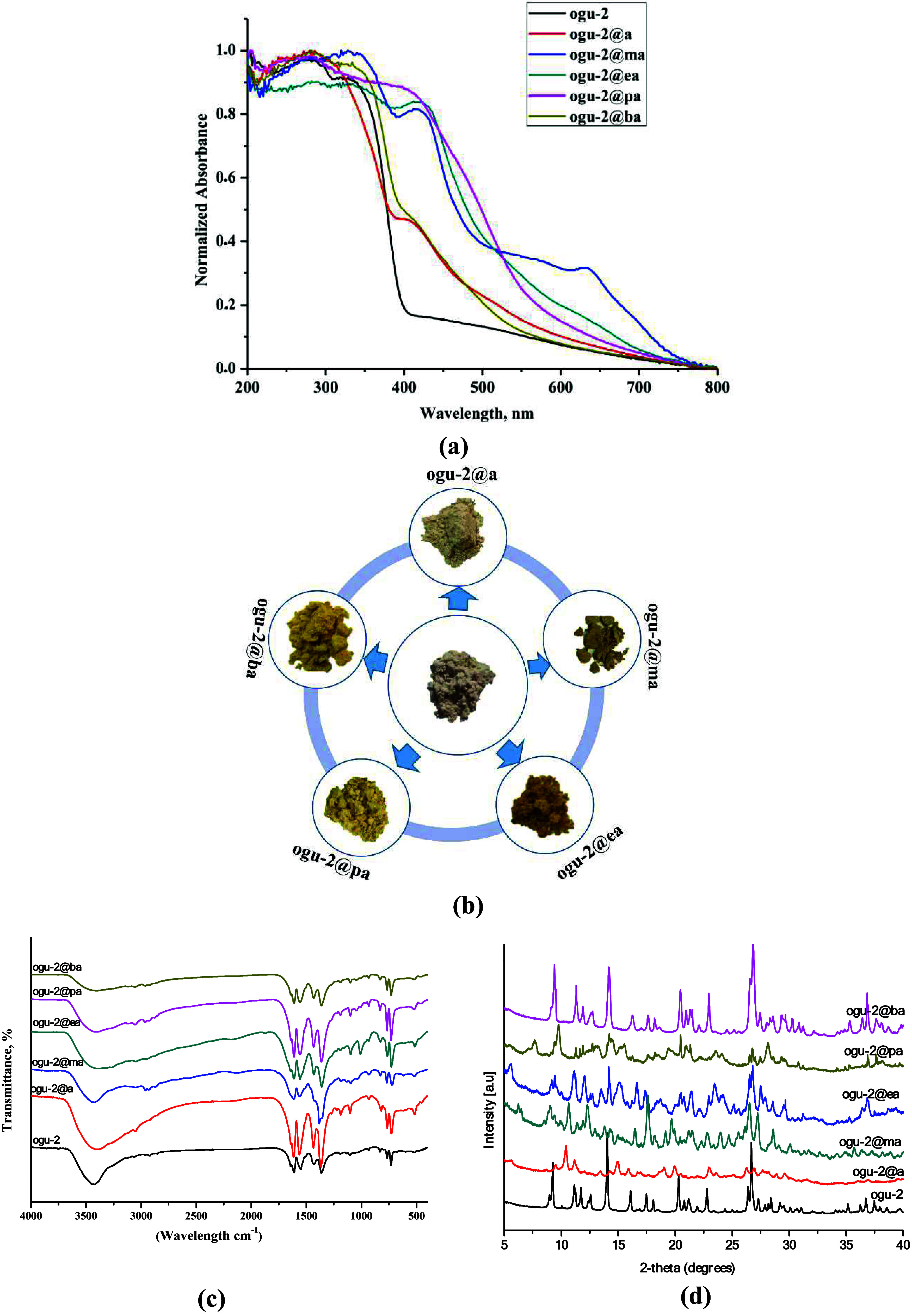
(a) Diffuse reflectance spectra and (b) photographs of **OGU-2** before and after treating with different amines (a = amin, ea = ethylamine, ma = methylamine, pa = propylamine, ba = butylamine). (c) FT-IR spectra and (d) PXRD spectra.

When examining similar studies in the literature, it can be seen that electron-deficient viologen compounds react with electron-rich volatile compounds by undergoing color changes. Therefore, the vapochromic behavior of **OGU-2**, in response to volatile amines (such as ammonia, methylamine, ethylamine, propylamine, and butylamine), was also investigated. As shown in Figure 4, **OGU-2**^**b**^ exhibited distinct and varied color changes upon exposure to different ammonia vapors. This behavior shows that different volatile organic amines can be easily distinguished using **OGU-2**.

**OGU-2** exhibits no significant color change upon exposure to ammonia vapor, whereas exposure to methylamine vapor induces greenish hues. In contrast, ethylamine, propylamine, and butylamine vapors lead to yellowish color shifts. Furthermore, the intensity of these color changes increased proportionally with the concentration of amine vapor, reaching saturation after 1 h of exposure (Figure S3). To further confirm the structural integrity of **OGU-2**^**b**^ following its interaction with different amine compounds, PXRD and FT-IR analyses were performed (Figure 4). FT-IR analysis of **OGU-2**^**b**^ samples exposed to amine vapors showed the emergence of new peaks corresponding to amine derivatives (Figure 4c). Furthermore, a comparison of the PXRD patterns of **OGU-2** and **OGU-2**^**b**^ samples showed no significant differences, indicating that the chemical structure of **OGU-2**^**b**^ remained unchanged (Figure 4d). This observation indicates that electron transfer (ET) occurred from the electron-rich nitrogen atoms of the volatile amine compounds to the electron-deficient nitrogen atoms within the viologen structure, leading to the formation of free radicals. As is well known, the lone electron pairs on amine compounds possess higher energy levels compared to those on oxygen atoms. Consequently, this electron transfer does not require UV–vis light excitation and can occur at room temperature under white light. Thus, viologen-based **OGU-2** represents a promising candidate for the naked eye detection of volatile organic amines (Figure 4).

### 3.4 QR Code Application

**Figure 5:**
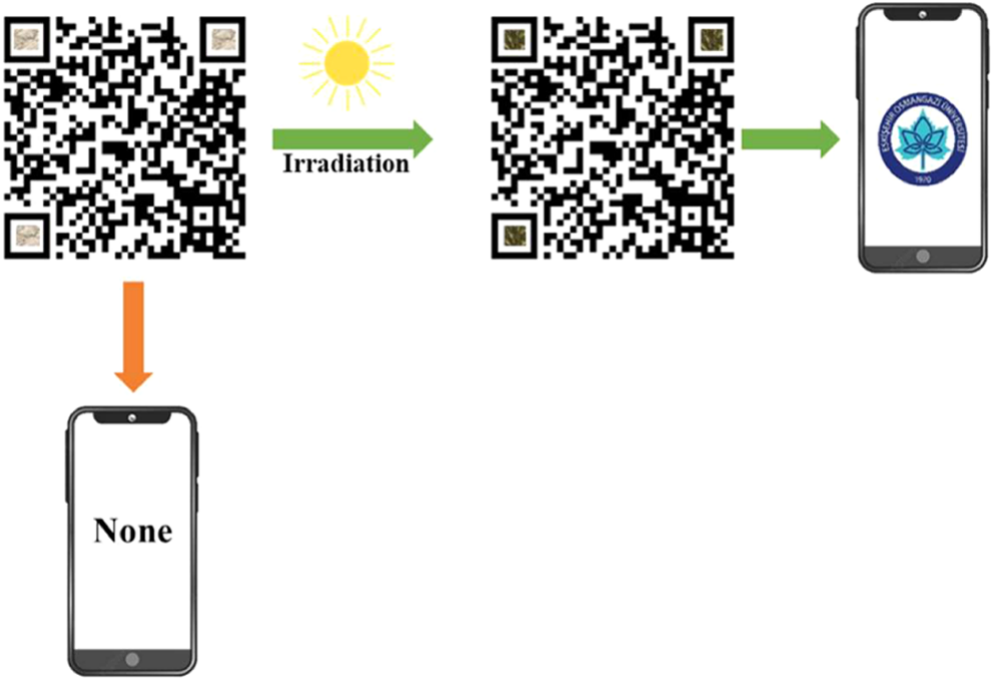
Schematic of the coloring process of **OGU-2** as a material for QR code anticounterfeiting.

**OGU-2** has been investigated as a material for counterfeiting protection due to its rapid photochromic response. A small amount of ethanol was added to finely ground **OGU-2**, and the mixture was sonicated for 30 min. After printing the QR code, the resulting suspension was evenly applied to the corners of the selected QR code and allowed to air-dry. As shown in Figure 5, the **OGU-2** could not be read by a smartphone due to its light color before exposure to light. However, when exposed to a Xe lamp for 5 s or to the light of the smartphone for 30 s, the **OGU-2** turns dark green, allowing the smartphone to read the QR code. This demonstrates the potential application of **OGU-2** in the field of anticounterfeiting.

### 3.5 Thermal Analysis and PXRD

Thermogravimetric analysis (TA) and powder X-ray diffraction (PXRD) were performed to evaluate the stability and phase purity of **OGU-2**. The thermal stability and decomposition mechanism of **OGU-2** were analyzed using thermal analysis methods (TG, DTG, and DTA). The endothermic peak observed between 35 and 110 °C corresponds to the loss of three crystal water molecules. Upon further heating between 360 and 512 °C, the decomposition of btc and Me_2_bipy occurs exothermically. The final thermal decomposition product is CdO (Figure S4).

Powder X-ray diffraction (PXRD) analysis was performed to evaluate the phase purity and structural integrity of **CoMOF** and **OGU-2**. The theoretical diffraction pattern was simulated using the Mercury program based on single-crystal X-ray data. A detailed comparison of the simulated and experimental PXRD patterns, recorded both before and after UV irradiation, demonstrated a high degree of consistency. These findings confirmed that the complex retained its phase purity and structural stability, with no significant changes observed in the diffraction peaks after irradiation (Figure 2b).

### 3.6 Hydrogen Production

#### Effect of NaBH_4_ Amount

**Figure 6:**
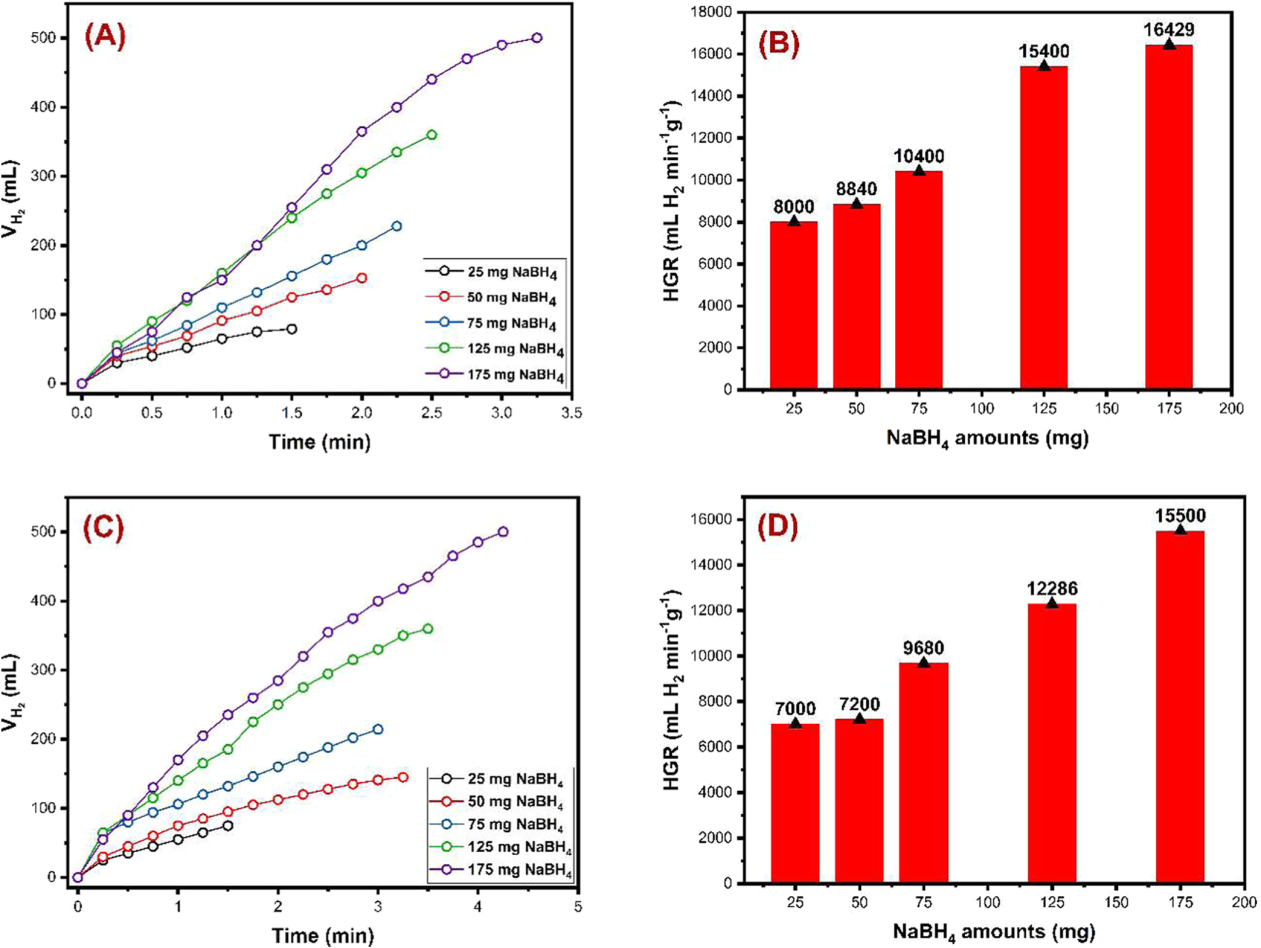
Effect of the NaBH_4_ amount on hydrogen production for **OGU-2** and **Co-MOF**. (A, C) Time vs volume of hydrogen plots, respectively, and (B, D) amount of NaBH_4_ vs HGR (10 mg catalyst, 25–175 mg NaBH_4_, 10 mL MeOH, 30 °C, and 750 rpm mixing rate), respectively.

The amount of NaBH_4_ used in methanolysis reactions significantly influences both the volume of H_2_ gas produced and the reaction time. Therefore, this study investigated the effect of varying NaBH_4_ amounts. Experiments were conducted using NaBH_4_ amounts of 25, 50, 75, 125, and 175 mg (Figure 6), while maintaining constant conditions of 10 mL of MeOH, 10 mg of catalyst, 30 °C, and a stirring speed of 750 rpm. The results of the methanolysis reactions are presented in Table 1. For catalysts **OGU-2** and **Co-MOF**, hydrogen production times were observed to range between 1.5–3.25 and 1.5–4.25 min, respectively, depending on the amount of NaBH_4_. The hydrogen generation rate (HGR) values were calculated to be in the range of 8000–16,429 mL of H_2_ min^–1^ g_cat_^–1^ for catalyst **OGU-2** and 7000–15,500 mL of H_2_ min^–1^ g_cat_^–1^ for catalyst **Co-MOF**. As anticipated, the HGR (mL of H_2_ min^–1^ g_cat_^–1^) increased with the amount of NaBH_4_ for both catalysts. Notably, catalyst **OGU-2** exhibited superior catalytic activity compared to **Co-MOF**. Specifically, the required volume of H_2_ gas was produced more rapidly with catalyst **OGU-2**, with production times consistently below 3 min, whereas H_2_ production times with catalyst **Co-MOF** exceeded 3 min. These findings conclusively demonstrate that catalyst **OGU-2** exhibits significantly better catalytic performance than catalyst **Co-MOF**.

**Table 1:** Hydrogen Production Times and HGRs for **OGU-2**, and **Co-MOF** with Different Parameters

		hydrogen production time (min)		HGR (mL of H_2_ min^–1^ g_cat_^–1^)	
NaBH_4_ amount (mg)	*T* (°C)	**OGU-2**	**Co-MOF**	**OGU-2**	**Co-MOF**
25	30	1.5	1.5	8000	7000
50		2	3.25	8840	7200
75		2.25	3	10,400	9680
125		2.5	3.5	15,400	12,286
175		3.25	4.25	16,429	15,500
125	30	2.5	3.5	15,400	12,286
	40	2.25	3	17,000	16,071
	50	1.5	2.75	27,400	23,000

#### Effect of Temperature

**Figure 7:**
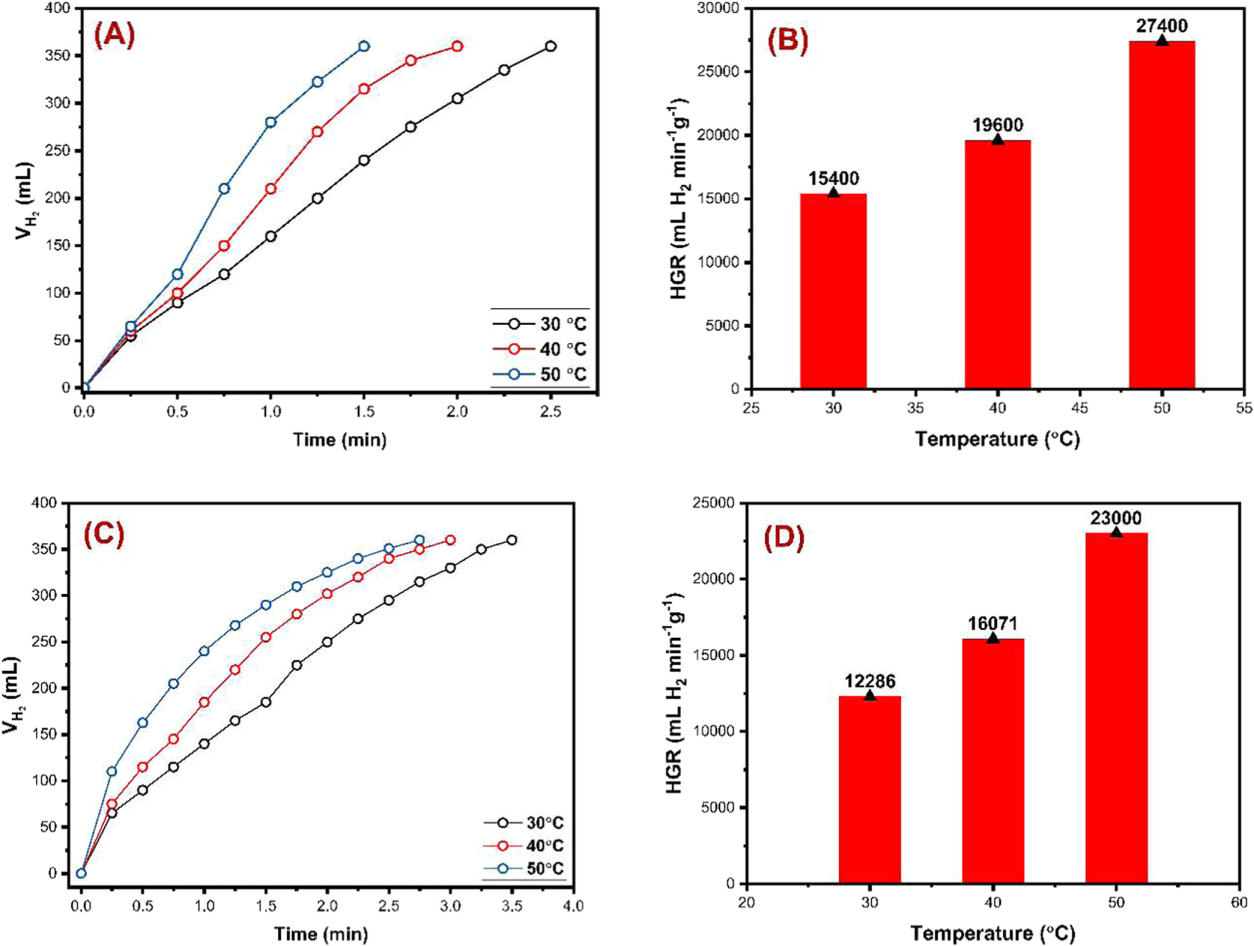
Effect of the temperature on hydrogen production for **OGU-2** and **Co-MOF**. (A, C) Time vs volume of hydrogen plots, respectively, and (B, D) temperature vs HGR (10 mg catalyst, 125 mg NaBH_4_, 10 mL MeOH, 30–50 °C, and 750 rpm mixing rate), respectively.

Temperature is a critical factor influencing the rate of most chemical reactions. Its impact is particularly significant in hydrogen (H_2_) production via the methanolysis of NaBH_4_. In this study, methanolysis reactions were performed at three different temperatures: 30, 40, and 50 °C. The experimental conditions were kept constant (10 mg of catalyst, 125 mg of NaBH_4_, 10 mL of MeOH, and a stirring speed of 750 rpm). The results, as presented in Table 1 and Figure 7, reveal that H_2_ production times for **OGU-2** and **Co-MOF** ranged from 1.5 to 2.5 min and from 2.75 to 3.5 min, respectively, depending on the temperature. HGR values were calculated to range from 15400 to 27400 mL of H_2_ min^–1^ g_cat_^–1^ for **OGU-2** and from 12286 to 23000 mL of H_2_ min^–1^ g_cat_^–1^ for **Co-MOF**. The catalytic activity of **OGU-2** increased by 10.4% when the temperature rose from 30 to 40 °C, and by 61.2% from 40 to 50 °C. For **Co-MOF**, the catalytic activity increased by 30.8% between 30 and 40 °C, and by 43.1% between 40 and 50 °C. Overall, 20 °C temperature increase enhanced the catalytic activity of **OGU-2** by 77.9% and **Co-MOF** by 87.2%. Although the performance improvement with increasing temperature was more pronounced for **Co-MOF**, H_2_ gas production occurred in a shorter time with **OGU-2**. As anticipated, the catalytic activity of both catalysts increased with rising temperatures, consistent with trends observed in previous studies.^23,30,31^ Notably, the highest HGR value of 27,400 mL of H_2_ min^–1^ g_cat_^–1^ was achieved with **OGU-2**.

**Figure 8:**
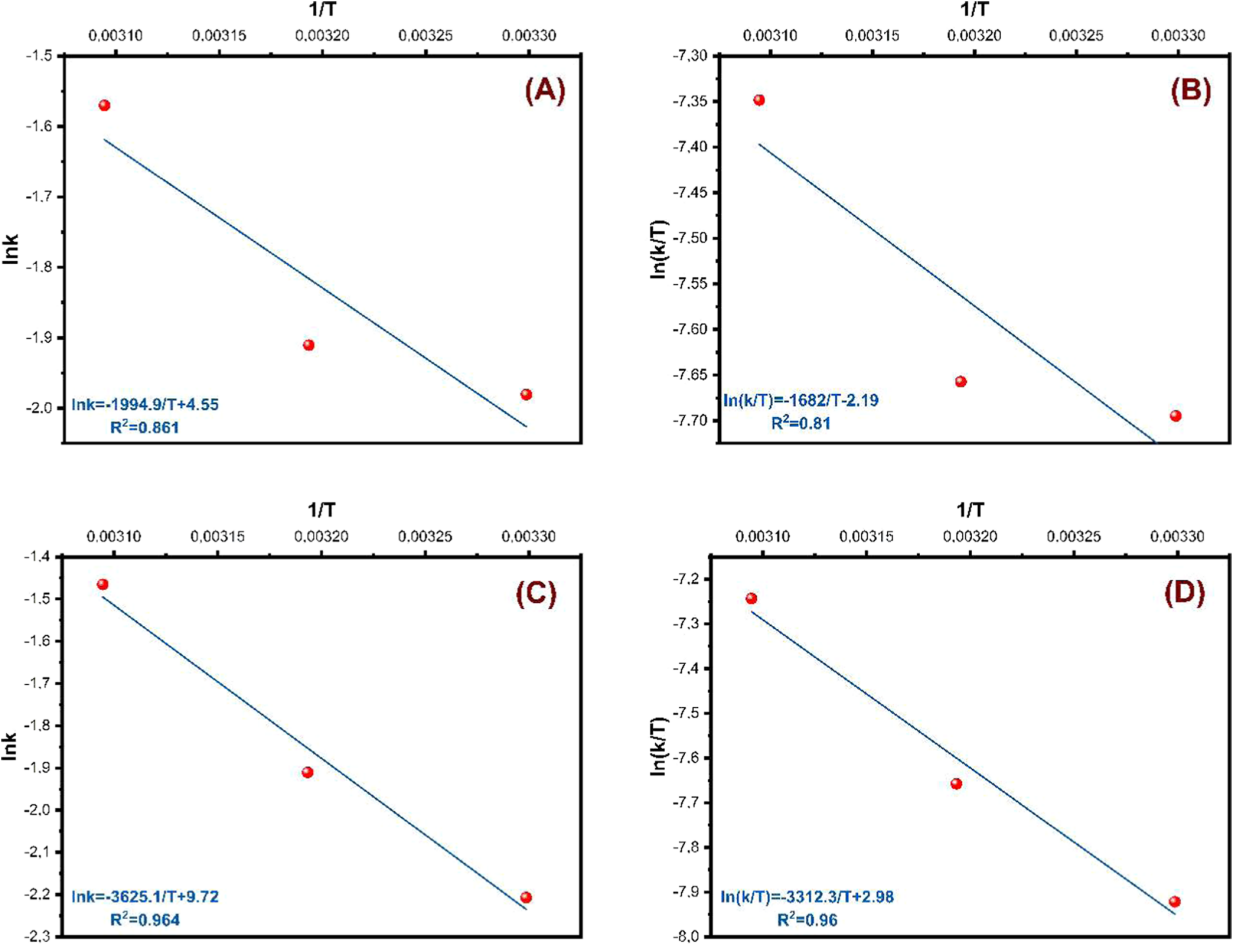
For **OGU-2** and **Co-MOF**, (A, C) ln *k* vs 1/*T* plots, respectively, and (B, D) ln *k*/*T* vs 1/*T* plots, respectively (30–50 °C, 10 mg catalyst, 125 mg NaBH_4_, 10 mL MeOH, and 750 rpm mixing rate).

According to the Arrhenius equation (eqs 1 and 2), the rate of a reaction increases with increasing temperature:
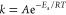
1
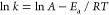
2In these equations, *k* is the rate constant, *E*_a_ is the activation energy, *T* is the temperature (K), *R* is the gas constant (8.314 J mol^–1^ K^–1^), and *A* is the frequency factor. The slope of the ln* k* versus 1/*T* graph drawn with eq 2 gives the activation energy. Accordingly, the ln *k* versus 1/*T* graphs drawn for **OGU-2** and **Co-MOF** are given in Figure 8A,C, and the *E*_a_ values calculated from the slopes of these graphs are given in Table 2. The *E*_a_ values for **OGU-2** and **Co-MOF** were calculated as 16.60 and 30.14 kJ mol^–1^, respectively.

**Table 2:** Values of *E*_a_, Δ*H*^#^, and Δ*S*^#^ for NaBH_4_ Methanolysis Reaction (10 mg Catalyst, 125 mg NaBH_4_, 10 mL MeOH, 30–50 °C and 750 rpm Mixing Rate)

catalyst	Δ*H*^#^ (kJ mol^–1^)	Δ*S*^#^ (J mol^–1^ K^–1^)	*E*_a_ (kJ mol^–1^)
**OGU-2**	13.98	–116.7	16.60
**Co-MOF**	27.54	–172.8	30.14

Also, Δ*H*^#^ (activation enthalpy) and Δ*S*^#^ (activation entropy) values can be calculated with the Eyring equation (eq 3):
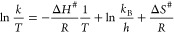
3In this equation, *k*_B_ is the Boltzmann constant (1.381 × 10^–23^ J/K) and *h* is the Planck constant (6.626 × 10^–34^ J·s). For this reason, the ln* k*/*T* versus 1/*T* graphs in Figure 8B,D were drawn. The Δ*H*^#^ and Δ*S*^#^ values for **OGU-2** and **Co-MOF** were calculated from the slopes and intercepts and are shown in Table 2.

#### Hydrogen Production Mechanism

**Figure 9:**
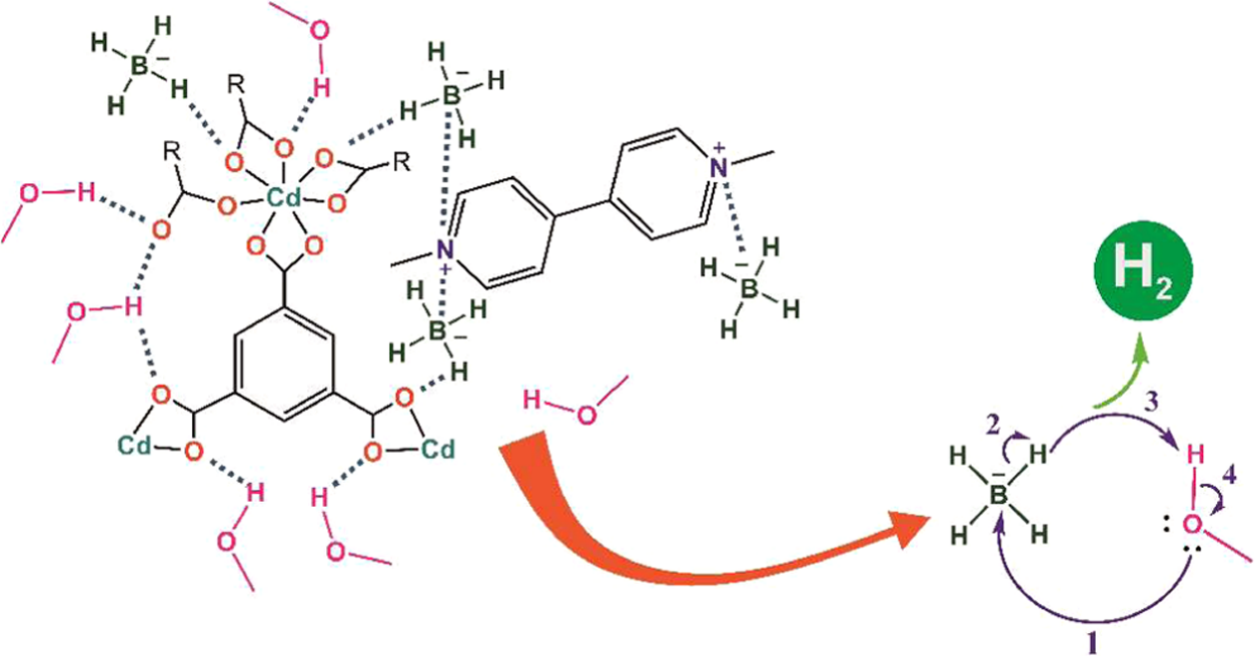
Possible interaction between catalysts, MeOH, and BH_4_^–^ and hydrogen production mechanism.

In order to understand the catalytic mechanism, first, the properties of the components in the reaction medium should be known. Methanol contains highly electronegative oxygens in its structure. Therefore, MeOH is a polar molecule and the electron density is high on the oxygen (it has a dipole moment). It is also well known that the −OH groups of MeOH make strong hydrogen bonds. Therefore, MeOH was adsorbed on the surface of **OGU-2** or **Co-MOF** in the reaction medium by strong hydrogen bonds or ion-dipole interactions. On the other hand, BH_4_^–^ is anionic and entered into ion–ion interactions with the quarternized nitrogens in the structure of **OGU-2** or **Co-MOF** during the reaction (Figure 9). In other words, **OGU-2** and **Co-MOF** rapidly adsorbed MeOH and BH_4_^–^ to their surfaces in the reaction medium and hydrogen production occurred as follows:

1. The oxygen atom of MeOH makes a nucleophilic attack on the B atom.
2. A hydride ion left from BH_4_^–^.
3. Hydride ion nucleophilically attacks the proton of the −OH group in MeOH and hydrogen gas is released

The strong adsorption ability of **OGU-2** and **Co-MOF** accelerated the production of hydrogen gas.

#### Comparison with the Literature

In order to understand the catalytic activities of **OGU-2** and **Co-MOF** more clearly, the obtained results were compared with other studies in the literature (Table 3). HGR (mL of H_2_ min^–1^ g_cat_^–1^) and *E*_a_ (kJ mol^–1^) values were taken into consideration in the comparison. **OGU-2** HGR and *E*_a_ values were determined as 15400 mL of H_2_ min^–1^ g_cat_^–1^ and 16.60 kJ mol^–1^, respectively. When Table 3 was examined in terms of HGR, it was seen that the values of 16250 and 20425 mL of H_2_ min^–1^ g_cat_^–1^ obtained with porous hazelnut shell^32^ and natural lignin,^33^ respectively, were higher than **OGU-2**. Porous hazelnut shell is an activated carbon, and according to FT-IR results, it contains C–O, N–O, and C=O groups. These groups form hydrogen bonds with NaBH_4_ and MeOH during the reaction. In addition, the surface area of porous hazelnut shell is 971 m^2^ g^–1^. Due to its high surface area and strong hydrogen bonding ability, the HGR values obtained for porous hazelnut shell were higher. These properties also caused porous hazelnut shells to have a lower *E*_a_ value (11.45 kJ mol^–1^). It was thought that the most dominant feature for porous hazelnut shell was the high surface area. The situation was different for natural lignin. Because the surface area of natural lignin was 15.15 m^2^ g^–1^, the determining factor for catalytic activity for lignin was its high amount of −OH, –OCH_3_, and ether functional groups. These groups increased the reaction rate by forming hydrogen bonds with NaBH_4_ and MeOH intensively. However, most probably due to its very low surface area, the *E*_a_ value of natural lignin was 19.5 kJ mol^–1^. As a result, it was clearly seen that **OGU-2** showed quite good catalytic activity compared to the literature. When the HGR and *E*_a_ values for **Co-MOF** (12286 mL of H_2_ min^–1^ g_cat_^–1^ and 30.14 kJ mol^–1^, respectively) were compared with Table 3, it was understood that **Co-MOF** showed a catalytic activity parallel to the literature. In terms of HGR, porous hazelnut shell,^32^ natural lignin,^33^ Cr_1.4_Na_0.6_O_3_,^34^ and Co–B catalysts^35^ have better HGR values than **Co-MOF**. In this respect, **Co-MOF** has high catalytic activity, while it has an average value in terms of *E*_a_.

**Table 3:** HGR and *E*_a_ Values of Various Polymer Catalysts with Methanolysis Method in the Literature

catalyst	*T* (°C)	HGR (mL of H_2_ min^–1^ g_cat_^–1^)	*E*_a_ (kJ mol^–1^)	ref
Co–Mo–P/CNTs-Ni foam	25	2642	47.27	(36)
P(C_6_VImBr)	25	5871	34.30	(37)
porous hazelnut shell	30	16,250	11.45	(32)
Co–B catalyst loaded with *C. vulgaris* microalgal strain cont. cellulose	30	13,215	25.22	(35)
AAPC–WBTL	45	7676	47.06	(38)
AAPC–WGTL	45	9084	47.89	
CAP	25	8182	34.8	(39)
H-EDA-CS	25	3460	26.14	(40)
natural lignin	25	20,425	19.5	(33)
carbonnanodods		1066	22.01	(41)
microgel (dextran + NH_2_S)	25	3175	30.72	(22)
modified mMWCNTs	25	8766	20.1	(42)
poly(caster oil) organoparticles	30	527.1	24	(43)
polymeric microgels	25	3018	27.96	(19)
chitosan-coated cellulose cotton fibers	22	7760	14.41	(44)
metal nanoparticle-doped cellulose cotton microfibers	22	2049	20.11	(30)
PDMA cryogel beads	30	4800	19.34	(45)
Cr_1,4_Na_0,6_O_3_	30	19,144		(34)
ZIF-67@GO-2	0	3200		(46)
ZnSnO_3_/SnO_2_	25	374.11	43.19	(47)
ZnONPs	25	308.5	17.45	(48)
OGU-2	30	15,400	16.60	In this study
Co-MOF	30	12,286	30.14	In this study

When **OGU-2** and **Co-MOF** were compared, it was clearly seen that **OGU-2** exhibited a better catalytic performance. The most fundamental difference between the two compounds was that they contained different metals in their structures (**OGU-2** and **Co-MOF** had Cd, and Co, respectively). Cadmium is larger than Co and since it is a less redox-active metal, it generally acts as a Lewis acid in catalytic processes. In other words, Cd may have facilitated the reactions required for the formation of H_2_ gas by polarizing NaBH_4_ molecules. Cobalt is generally a more redox-active element than Cd. This may provide an advantage in the electron transfer mechanism. However, the redox ability of the Co atom was most likely better stabilized by the ligands around it (especially oxygen and nitrogen atoms). As a result, the catalytic effect of **Co-MOF** was weaker than **OGU-2**.

## Conclusions

In this study, it was aimed to reveal highly effective catalysts that will accelerate the hydrogen production reaction through NaBH_4_ methanolysis, and the effectiveness of the synthesized **OGU-2**, and **Co-MOF** catalysts. Experiments on H_2_ gas production from NaBH_4_ via methanolysis were carried out at 30 °C, with 125 mg NaBH_4_ and 10 mg catalyst amounts, 10 mL of MeOH, and 750 rpm stirring speed. Under these conditions, the required H_2_ gas volumes of **OGU-2** and **Co-MOF** were produced in 2.5 and 3.5 min, respectively. Depending on this time and the produced H_2_ gas volume, HGR values were calculated as 15400 and 12286 mL of H_2_ min^–1^ g_cat_^–1^, respectively. When compared with the literature, it was seen that **OGU-2** and **Co-MOF** had quite high catalytic performance.

## Supporting Information Available

FT-IR spectra for **Co-MOF** and **OGU-2** (Figure S1); PXRD spectra for **Co-MOF** (Figure S2); color change of amine derivatives after timed exposure of **OGU-2** (Figure S3); thermal analyses curves of **OGU-2** (Figure S4); time-dependent photochromic response of **OGU-2** upon sunlight exposure (Figure S5); diffuse reflectance spectra of **OGU-2** (Figure S6); fluorescence spectra of OGU-2 before and after exposure to sunlight for 15 minutes ( Figure S7); crystal data and structure parameters for **OGU-2** (Table S1); bond lengths and bond angles of **OGU-2** (Table S2); photoresponsive times and photochromic behavior of various coordination polymers (Table S3) (PDF)
