# Supplementary material for: Cobalt(II) and Cadmium(II) Metal–Organic Framework with Benzene-1,3,5-tricarboxylate and Viologen Guest: Stimuli-Responsive Photochromism, Volatile Amine Detection, and Hydrogen Generation
Source: ACS Omega. 2025 Apr 17;10(16):16938–51. doi: 10.1021/acsomega.5c01484 (PMC12044445; doi:10.1021/acsomega.5c01484)
Supplement: Supplementary file 1 — ao5c01484_si_001.pdf [file ao5c01484_si_001.pdf]

Cobalt(II) and Cadmium(II) Metal-Organic Framework with Benzene-1,3,5-Tricarboxylate and Viologen Guest: Stimuli-Responsive Photochromism, Volatile Amine Detection and Hydrogen Generation

Ferihan Tataş Coşkun[a], Kutalmış Gökkuş[b] and Okan Zafer Yeşilel[a,*]

[a]Eskişehir Osmangazi University, Department of Chemistry, Faculty of Science, 26480 Eskişehir, Türkiye

[b]Kastamonu University, Department of Environmental Engineering Faculty of Engineering and Architecture, 37500 Kastamonu, Türkiye

Fig. S1. FT-IR spectra for (a) **Co-MOF** and (b) **OGU-2**

Fig. S2. PXRD spectra for **Co-MOF**

Fig. S3. Color change of amine derivatives after timed exposure of **OGU-2**

Fig. S4. Thermal analyses curves of **OGU-2**

Fig. S5. Time-dependent photochromic response of **OGU-2** upon sunlight exposure. The structural representation illustrates the gradual color change from orange to blue over a period of 15 minutes.

Fig. S6. (a) Diffuse Reflectance Spectra of **OGU-2** before and after exposure to sunlight for 15 minutes.

Table S1. Crystal data and structure parameters for **OGU-2**

Tablo S2. Bond lengths (Å) and bond angles (°) of **OGU-2**

Table S3. Photoresponsive times and photochromic behavior of various coordination polymers

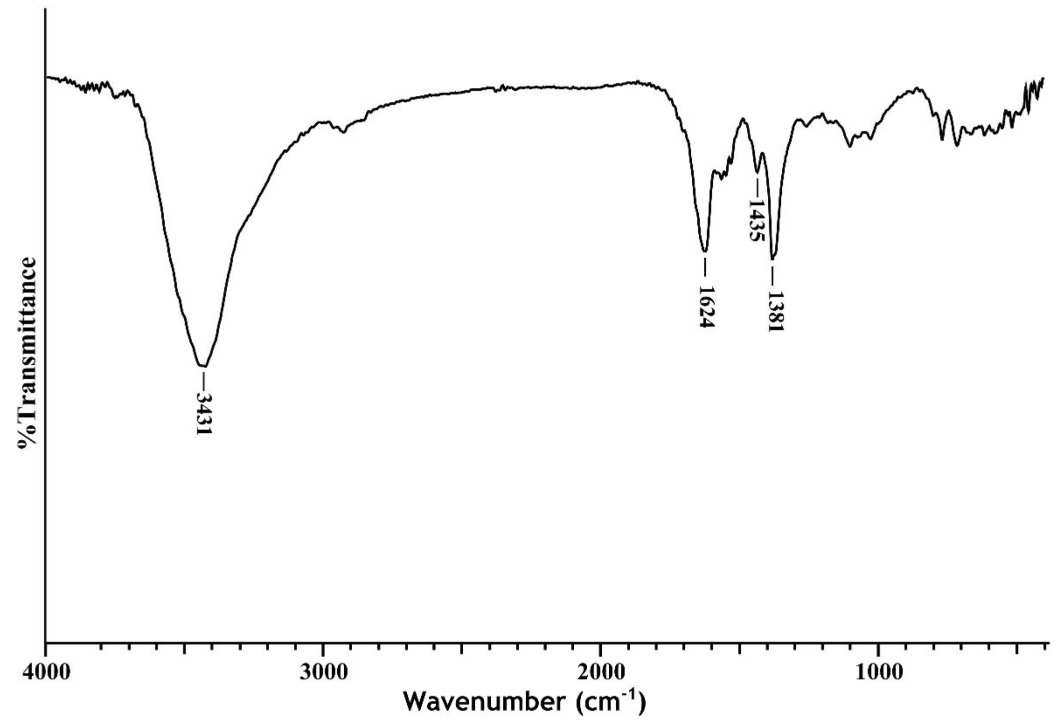

(a)

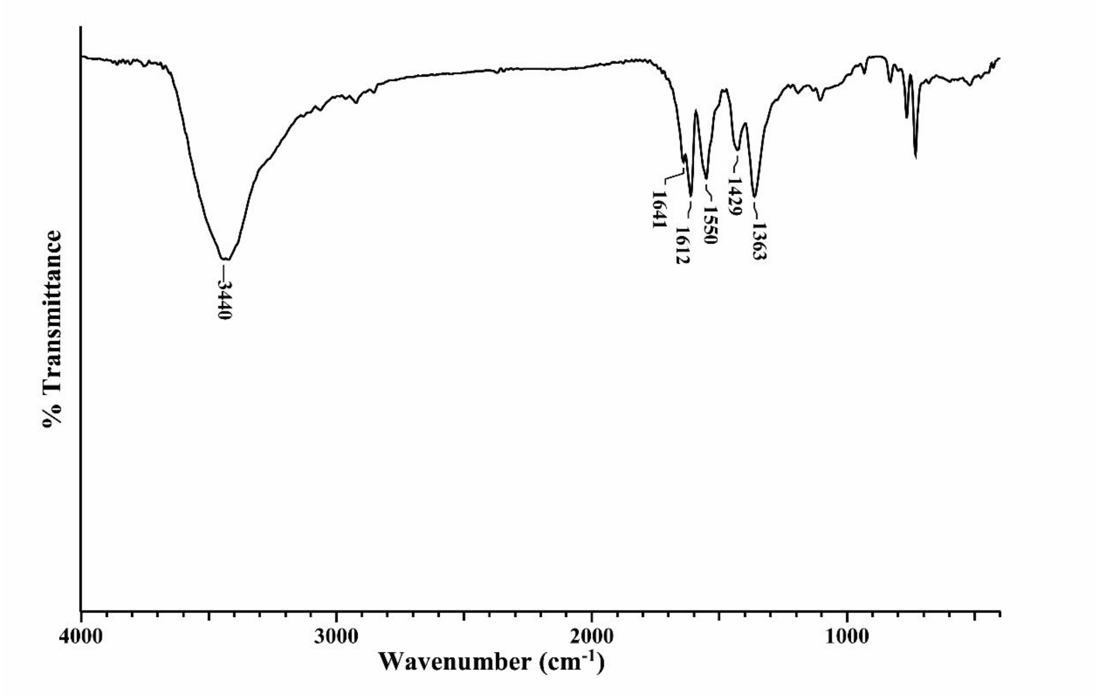

(b)

Fig. S1. FT-IR spectra for (a) **Co-MOF** and (b) **OGU-2**

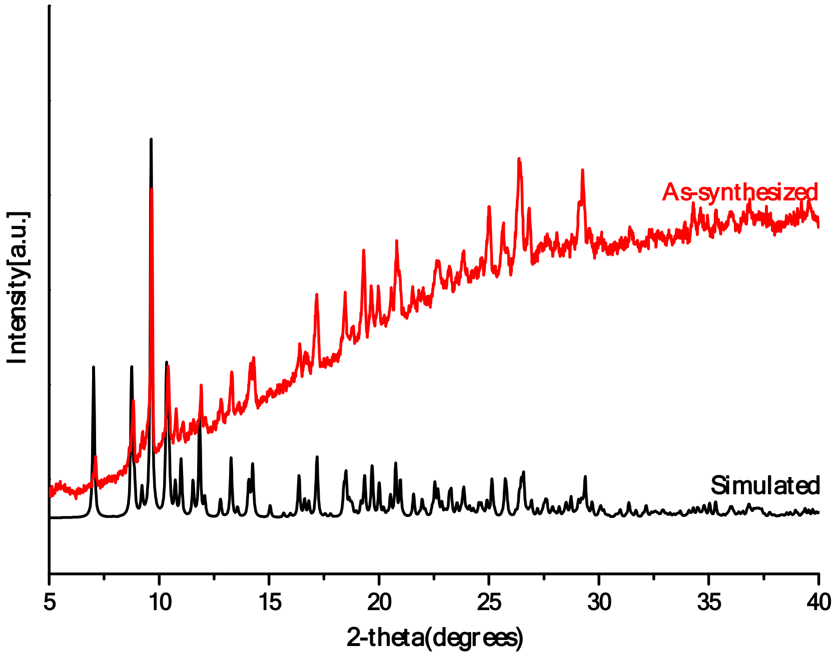

Fig. S2. PXRD spectra for **Co-MOF**

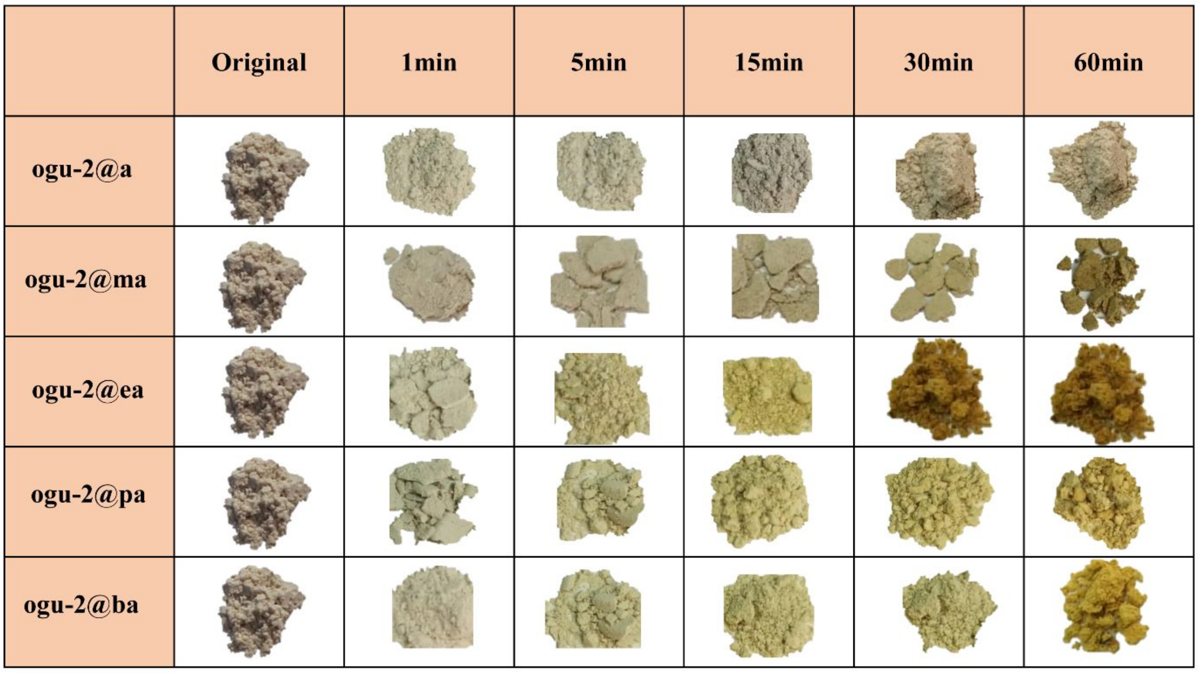

Fig. S3. Color change of amine derivatives after timed exposure of **OGU-2**

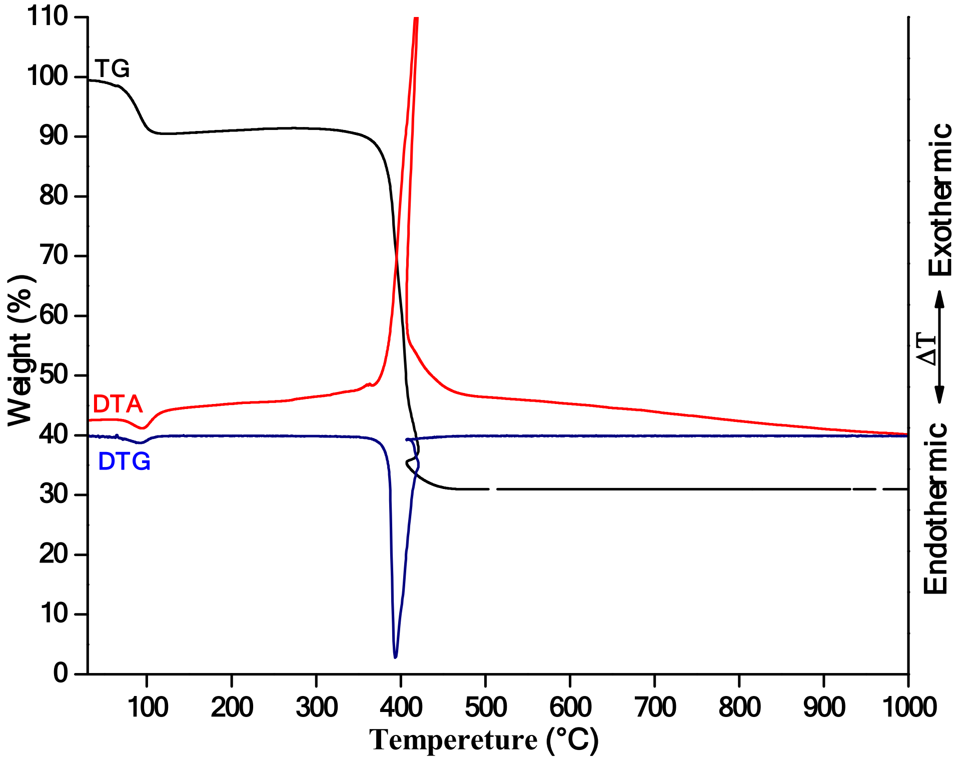

Fig. S4. Thermal analyses curves of **OGU-2**

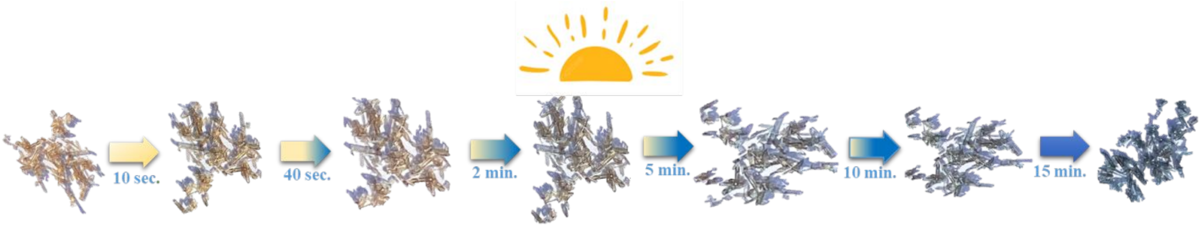

Fig. S5. Time-dependent photochromic response of **OGU-2** upon sunlight exposure. The structural representation illustrates the gradual color change from orange to green over a period of 15 minutes.

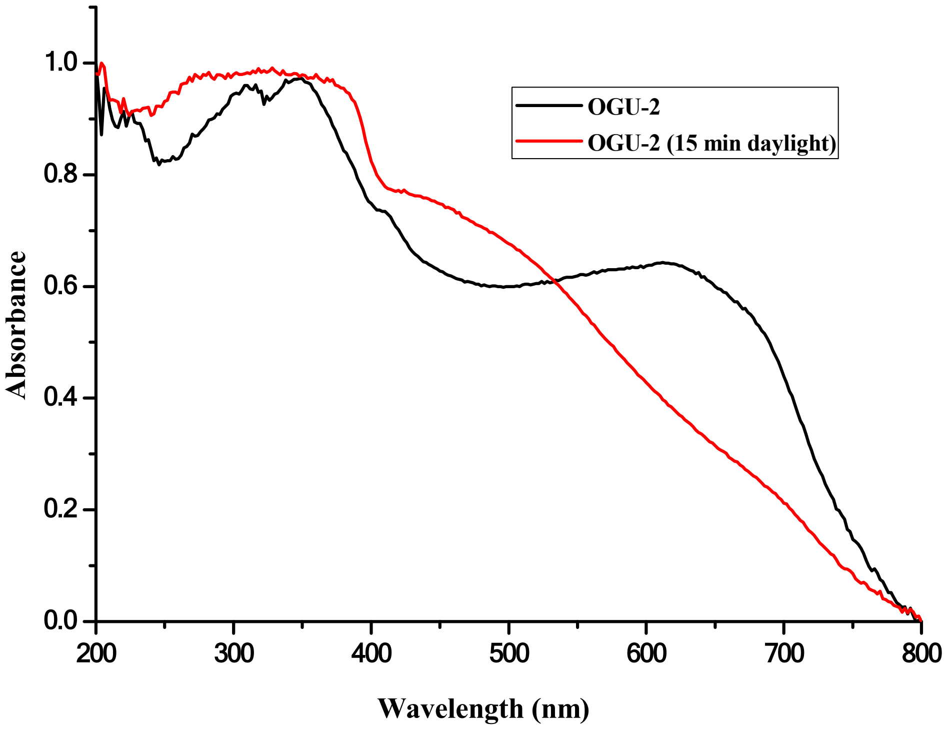

Fig. S6. Diffuse Reflectance Spectra of **OGU-2** before and after exposure to sunlight for 15 minutes

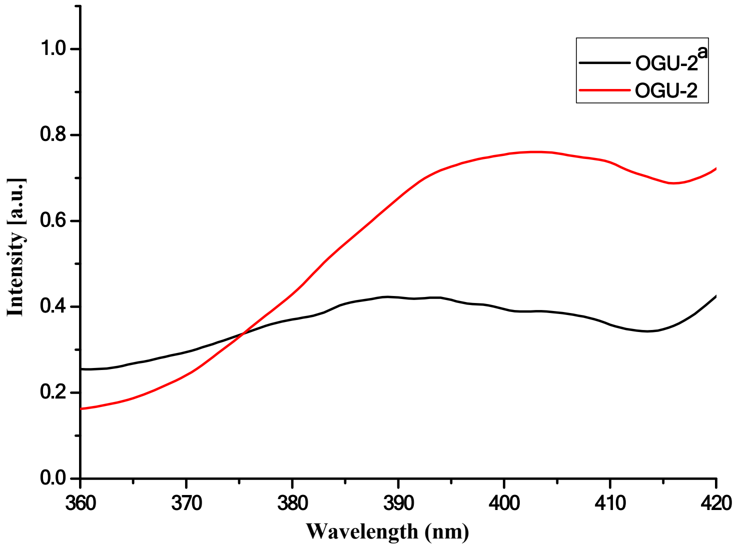

Fig. S7. Fluorescence spectra of **OGU-2** before and after exposure to sunlight for 15 minutes

Table S1. Crystal data and structure parameters for **OGU-2**

**Compounds**	**OGU-2**
**Formula**	$C_{15}H_{18}NO_{10}Cd$
**MW (gmol^{-1})**	484.70
**Diffractometer**	Bruker APEX II CCD
**Rad. /λ (Å)**	Mo-K_{α} / 0.71073
**Temperature (K)**	273
**Colour**	Brown
**Crystal System**	Monoclinic
**Space Group**	*C*2/c
**a (Å)**	13.2843 (7)
**b (Å)**	15.5809 (8)
**c (Å)**	16.6935 (11)
**α (º)**	90
**β (º)**	109.396 (2)
**γ (º)**	90
**V (Å^3)**	3259.1 (3
**Z**	8
**d (g cm^{-3})**	1.976
**θ (º)**	2.6–28.4
**$R_{int.}$**	0.046
**$R[F^2 > 2\sigma(F^2)]$**	0.096
**$wR(F^2)$**	0.194
**S**	1.09
**$\Delta\rho_{max}/\Delta\rho_{min}$ (e Å^{-3})**	4.14/–6.21

Tablo S2. Bond lengths (Å) and bond angles (º) of **OGU-2**

**Bond Lengths (Å)**			
Cd1–O1	2.273 (5)	Cd1–O4^{i}	2.504 (6)
Cd1–O2	2.611 (6)	Cd1–O5^{iii}	2.250 (6)
Cd1–O1^{ii}	2.518 (6)	Cd1–O6^{iii}	2.592 (7)
Cd1–O3^{i}	2.247 (5)		
**Bond Angles (º)**			
O1–Cd1–O2	52.92 (19)	O3^{i}–Cd1–O2	89.3 (2)
O1–Cd1–O1^{ii}	72.0 (2)	O4^{i}–Cd1–O1^{ii}	142.37 (19)
O1–Cd1–O4^{i}	131.9 (2)	O4^{i}–Cd1–O6^{iii}	77.3 (2)
O1–Cd1–O6^{iii}	144.2 (2)	O4i–Cd1–O2	80.57 (19)
O1^{ii}–Cd1–O6^{iii}	96.9 (2)	O5^{iii}–Cd1–O4^{i}	115.3 (2)
O1^{ii}–Cd1–O2	106.7 (2)	O5^{iii}–Cd1–O1	91.9 (2)
O3^{i}–Cd1–O4^{i}	55.05 (19)	O5^{iii}–Cd1–O1^{ii}	87.6 (2)
O3^{i}–Cd1–O1^{ii}	87.7 (2)	O5^{iii}–Cd1–O6^{iii}	52.9 (2)
O3^{i}–Cd1–O1	126.1 (2)	O5^{iii}–Cd1–O2	132.1 (2)
O3^{i}–Cd1–O5^{iii}	137.6 (2)	O6^{iii}–Cd1–O2	155.8 (2)
O3^{i}–Cd1–O6^{iii}	86.0 (2)		
**Symmetry codes:** x+½, -y+3/2, z+½; (ii) -x+3/2, -y+3/2, -z+1; (iii) x+½, y-½, z; (iv) -x+3/2, -y+3/2, -z+2; (v) x-½, -y+3/2, z-½; (vi) x-½, y+½, z			

Table S3. Photoresponsive times and photochromic behavior of various coordination polymers

Compounds	Photoresponsive time	Photochromic behavior	Ref.
$\{[Cd_2(L)(PTA)_2]\cdot 3H_2O\}_n$	30 min	Yellow → Deep blue	**1**
$[Zn_3(Hbdpd)(bdpd)(Cl)(Bbpy)]$	20 min	Yellow → Green	**2**
$\{[Zn(CEbpy)_2(H_2O)_2][Zn(p\text{-}BDC)(H_2O)_4]_2Br\}_n$	10 min	Light Brown → Deep purple	**3**
$\{[Cd(CEbpy)(\mu\text{-}BDC)(DMF)]\cdot 2H_2O\}_n$	2 min	Yellow→ Dark blue	**4**
$\{[Zn(H_2O)_6][Zn(BTEC)(H_2O)_4](bcbpy)\cdot 4H_2O\}_n$	1 min	Colorless → Dark blue	**5**
$[ZnL]\cdot 2H_2O$	1 min	Yellow → Brownish red	**6**
$[Zn(bpdo)(fum)(H_2O)_2]_n$	1 min	Yellowish → Green	**7**
**OGU-2** ($\{(Me_2bipy)[Cd_2(\mu_4\text{-}btc)_2]\}_n$)	10 sec	Orange → Green	**This work**
$[Cd_2(\mu_4\text{-}L)Cl_3]_n$	3 sec	White →Light blue	**8**

References

(1) Liu, J. P.; Zhang, C. R.; Li, L. K.; Zang, S. Q. A Multistimuli-Responsive Cd-MOF Constructed by an Extended Viologen-Based Carboxylate Ligand. *Cryst Growth Des* **2024**, *24* (8), 3299-3308. DOI: 10.1021/acs.cgd.4c00030.

(2) Chen, C. Y.; Liu, J. F.; Sun, Z.; Yang, S. J.; Yuan, S. W.; Han, H. T. A Zn (II)-viologen MOF material: Photochromic, photoswitchable luminescent properties and UV sensing. *J Mol Struct* **2023**, *1283*. DOI: ARTN 135297

10.1016/j.molstruc.2023.135297.

(3) Zhao, G. Z.; Liu, J. J. Two Zn-viologen coordination polymers constructed from 1-carboxyethyl-4,4′-bipyridinium ligands: Crystal structures, photochromism and theoretical calculations. *Polyhedron* **2021**, *205*. DOI: ARTN 115295

10.1016/j.poly.2021.115295.

(4) Liu, J. J.; Li, J.; Lu, W. B. Photochromic properties of three 2D MOFs based on 1-carboxyethyl-4,4′-bipyridinine. *Rsc Adv* **2019**, *9* (57), 33155-33162. DOI: 10.1039/c9ra06703e.

(5) Liu, J. J.; Lu, Y. W.; Lu, W. B. Metal-dependent photosensitivity of three isostructural 1D CPs based on the 1,1′-bis(3-carboxylatobenzyl)-4,4′-bipyridinium moiety. *Dalton T* **2020**, *49* (13), 4044-4049. DOI: 10.1039/d0dt00157k.
(6) Li, P.; Zhou, L. J.; Yang, N. N.; Sui, Q.; Gong, T.; Gao, E. Q. Metal-Organic Frameworks with Extended Viologen Units: Metal-Dependent Photochromism, Photomodulable Fluorescence, and Sensing Properties. *Cryst Growth Des* **2018**, *18* (11), 7191-7198. DOI: 10.1021/acs.cgd.8b01383.
(7) Zhang, N. N.; Xin, L. D.; Li, L.; Zhang, Y. N.; Wu, P. P.; Han, Y. F.; Yan, Y.; Qu, K. G. Multifunctional Crystalline Coordination Polymers Constructed from 4,4'-Bipyridine-N,N'-dioxide: Photochromism, White-Light Emission, and Photomagnetism. *Acs Omega* **2023**, *8* (37), 34017-34021. DOI: 10.1021/acsomega.3c04892.
(8) Luo, Y.; Liu, J. P.; Li, L. K.; Zang, S. Q. Multi-Stimuli-Responsive Chromic Behaviors of an All-in-One Viologen-Based Cd(II) Complex. *Inorg Chem* **2023**, *62* (35), 14385-14392. DOI: 10.1021/acs.inorgchem.3c02070.
